# Economic Evaluations Informed Exclusively by Real World Data: A Systematic Review

**DOI:** 10.3390/ijerph17041171

**Published:** 2020-02-12

**Authors:** Elizabeth Parody-Rúa, Maria Rubio-Valera, César Guevara-Cuellar, Ainhoa Gómez-Lumbreras, Marc Casajuana-Closas, Cristina Carbonell-Duacastella, Ignacio Aznar-Lou

**Affiliations:** 1 Teaching, Research & Innovation Unit, Parc Sanitari Sant Joan de Déu–Institut de Recerca Sant Joan de Déu, 08830 Barcelona, Spain; mrubio@pssjd.org (M.R.-V.); c.carbonell@pssjd.org (C.C.-D.); i.aznar@pssjd.org (I.A.-L.); 2 Primary Care Prevention and Health Promotion Network (redIAPP), 08007 Barcelona, Spain; 3 CIBER of Epidemiology and Public Health (CIBERESP), 28029 Madrid, Spain; 4 Faculty of Health Sciences, Universidad Icesi, 760030 Cali, Colombia; cguevara@icesi.edu.co; 5 Institut Universitari d’Investigació en Atenció Primària Jordi Gol (IDIAPJGol), 08007 Barcelona, Spain; agomez@idiapjgol.info (A.G.-L.); mcasajuana@idiapjgol.info (M.C.-C.); 6 Universitat Autònoma de Barcelona, 08193 Bellaterra (Cerdanyola del Vallès), Spain; 7 Health Science School, Universitat de Girona, 17071 Girona, Spain

**Keywords:** economic evaluation, systematic review, real world data, real world evidence, electronic health records

## Abstract

Economic evaluations using Real World Data (RWD) has been increasing in the very recent years, however, this source of information has several advantages and limitations. The aim of this review was to assess the quality of full economic evaluations (EE) developed using RWD. A systematic review was carried out through articles from the following databases: PubMed, Embase, Web of Science and Centre for Reviews and Dissemination. Included were studies that employed RWD for both costs and effectiveness. Methodological quality of the studies was assessed using the Consolidated Health Economic Evaluation Reporting Standards (CHEERS) checklist. Of the 14,011 studies identified, 93 were included. Roughly half of the studies were carried out in a hospital setting. The most frequently assessed illnesses were neoplasms while the most evaluated interventions were pharmacological. The main source of costs and effects of RWD were information systems. The most frequent clinical outcome was survival. Some 47% of studies met at least 80% of CHEERS criteria. Studies were conducted with samples of 100–1000 patients or more, were randomized, and those that reported bias controls were those that fulfilled most CHEERS criteria. In conclusion, fewer than half the studies met 80% of the CHEERS checklist criteria.

## 1. Introduction

Given limited health resources and growth of health technologies, it is vital to use these resources efficiently. Economic evaluation (EE) studies are a useful tool to facilitate decision-making regarding which health technologies to use and/or finance. EE studies can be performed directly, based on individual patient data during randomized controlled trials (RCT “piggyback”), or by employing data from pragmatic studies [1]. EE analyses can also be carried out using decision models. Studies that employ models can obtain data and parameters from RCTs, pragmatic studies, expert opinion or a combination of these sources. Other sources of data for EE are observational studies using Real World Data [RWD]. This information typology can be used to measure both effects and costs. As such, RWD can be used for decision models [2,3], pragmatic studies and retrospective observational studies. This review focuses on complete EE studies that use RWD but are not based on decision models. Although there are a large number of definitions of RWD, for the purposes of this review we refer to RWD as “Data used for decision-making that are not collected in conventional RCTs” [4,5]. Real World Evidence is generated from RWD to inform a conclusion or a judgment [6].

The main strengths of RWD are the large populations, which let us develop studies in low-prevalence diseases (unfeasible in the context of an RCT) and extensive time periods that allow examination of long-term effects. Consequently, and due to the use of software for management, storage and analysis of large data sets collected during daily clinical practice, use of RWD has been constantly increasing in recent years. The use of RWD is widely employed by Health Technology Agencies with several aims [7]. It is used for epidemiologic purposes to assess clinical effectiveness and safety of medications and other health technologies [8,9], for assessing adherence to and persistence with pharmacological treatments [10], to assess the direct and indirect costs of a certain treatment and/or procedures [11] and it is also considered a valid source for market access and reimbursement [2]. RWD also has some limitations: information considered as RWD is not always all the information we would like to achieve the research goals; some confounding factors might not be taken into account; RWD is susceptible to misclassification or omission as these data are collected during routine clinical practice with no research purpose as distinct from the use of the case report form (CRF) in clinical trials [12]. Once these limitations are addressed, the potential of using RWD to create evidence to guide improvements in health care systems will be notorious.

Developing economic evaluation using RWD will help in decision-making, optimizing resources and gaining external validity. Consequently, the main research goal of this paper is to review the characteristics and assess the quality of full economic evaluations developed using RWD worldwide. Our aim is to provide information to decision-makers on the methodological quality of the evaluated studies and identify the strengths and weaknesses with a view to improving such studies.

## 2. Materials and Methods

This was a systematic review of published studies. A protocol was drafted to conduct a systematic review following the recommendations of Preferred Reporting Items for Systematic Reviews and Meta-Analyses for systematic review protocols (PRISMA-P) (http://www.prisma-statement.org/Extensions/Protocols.aspx). [13].

**Protocol and registration**. The search protocol was registered at Figshare (https://figshare.com/articles/Systematic_Review_Protocol/7265576) in October 2018. The registration code is 7265576.

**Eligibility criteria**. Defined in PICO format in accordance with the aims of the present study (Population: any, Intervention: any, Comparator: any, Outcome: Full economic evaluation). Studies were considered complete economic evaluations if they compared two or more alternatives and assessed both costs and outcomes (effectiveness) and thus could be considered as cost-effectiveness analysis, cost-utility analysis and cost-benefit analysis [1]. There were no restrictions regarding date or publication status. Only studies in English or Spanish were included.

**Sources of information.** A search was performed of the main clinical databases: PubMed, Embase, Web of Science (WOS) and the Centre for Reviews and Dissemination (CRD). This search was carried out between the 3rd of June and the 21st of August 2018.

**Search strategy.** The search strategy was adapted to each of the databases. An example of the PubMed Search is provided in Supplementary Material 1.

**Inclusion criteria.** Complete economic evaluations that used RWD (data not collected in conventional RCT [5]) for both costs and effectiveness.

**Exclusion criteria**. Decision-Analytic Models, cost-minimization analysis, cost-analysis, reviews, meta-analysis, comments, protocols, use of ad-hoc questionnaires in costs or effects, poster or presentations at congress or workshops, grey literature, abstract only, not health-related, book or letter.

**Study selection.** Selection was conducted by independent pairs according to established inclusion and exclusion criteria. In cases of disagreement, a third reviewer assessed the article. Six researchers participated in the process.

All article titles and abstracts identified in the search were initially reviewed using the Rayyan QCRI platform (https://rayyan.qcri.org) [14]. This site allows identification of duplicated articles and establishes the degree of agreement between reviewer pairs.

Finally, those articles considered potentially eligible were fully reviewed by the reviewer pairs.

**Information extraction process.** Information extraction was performed independently in duplicate. The 6 researchers participated in the process. Study information was recorded in an Excel template with the following information: title, year of publication, country and study setting, year(s) of inclusion of the study population, sample size, illness of the population (Using the International Classification of Diseases, ICD-10 [15]), population-medication (for those studies including a population receiving a medication or group of medications, ATC [16] was/were recorded) population demographic data, intervention, control, study methodology, source of RWD, costs, outcomes, time horizon, perspective, risk of bias and methods for controlling it, and source of funding.

**Evaluation of methodological quality of included studies**. Assessment of the methodological quality of studies was conducted using the Consolidated Health Economic Evaluation Reporting Standards (CHEERS) checklist [17], ISPOR Task Force Report. This list contains 24 items for evaluation. Each item is assigned values from 0 to 2; where 0, represents non-compliance with the item evaluated, 1 represents compliance and 2 means not applicable. Not applicable is recorded in those items that refer to economic evaluation models (examples 15 and 16), as articles using economic decision models were excluded for the purposes of the study.

Study quality was also assessed by the independent pairs and differences were resolved through consensus. When consensus was not reached, a third reviewer was consulted. Selection of studies to be evaluated by each researcher was randomized.

Studies using the RWD checklist were not assessed [6] as some of the questions were included in the CHEERS list and, additionally, gave rise to ambiguities in interpretation by the different reviewers.

## 3. Results

**Figure 1:**
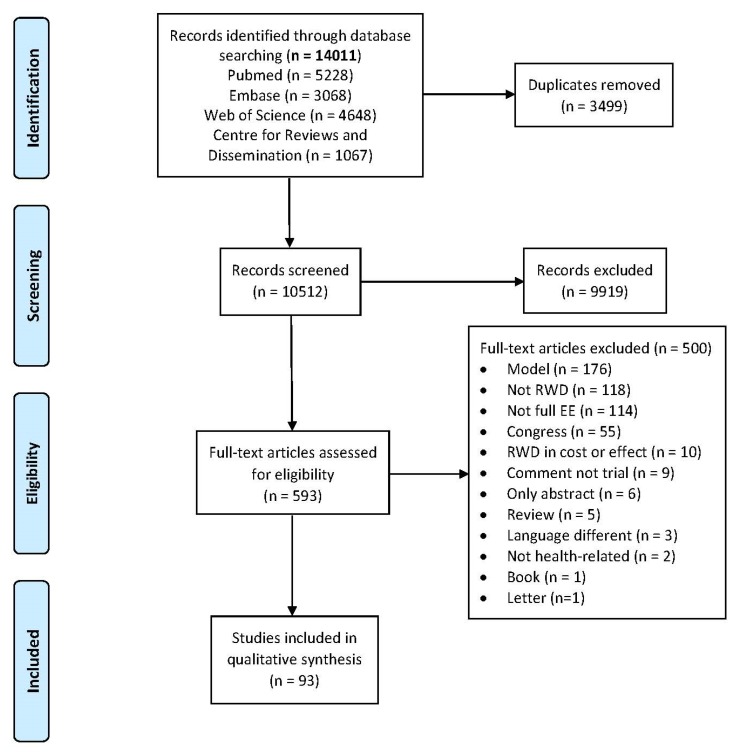
Flow chart of paper selection.

Of the 14,011 studies identified, 593 were potentially eligible following review of title and abstract. Figure 1 shows article selection and reasons for exclusion, mainly those studies using economic evaluation models, studies not using RWD or those that did not perform complete economic evaluations. A total of 93 articles were finally included [18,19,20,21,22,23,24,25,26,27,28,29,30,31,32,33,34,35,36,37,38,39,40,41,42,43,44,45,46,47,48,49,50,51,52,53,54,55,56,57,58,59,60,61,62,63,64,65,66,67,68,69,70,71,72,73,74,75,76,77,78,79,80,81,82,83,84,85,86,87,88,89,90,91,92,93,94,95,96,97,98,99,100,101,102,103,104,105,106,107,108,109,110].

Table 1 shows the characteristics of the included studies. Most were published between 2011 and 2018. The country with the most studies included was the USA (21.5%), while Europe provided 22 (notably France, Spain, Germany and United Kingdom). Sixteen studies did not report the country where it was carried out. Approximately 50% of studies performed the economic evaluation in a hospital setting and 29% did not specify the setting. Public funding was the type most frequently reported.

Regarding study population, the diseases most frequently assessed were neoplasms (14: chapter II), especially lung cancer and colorectal cancer (each representing 17% of the total of neoplasm studies), followed by breast cancer and hepatocellular cancer. Some 58% of studies did not report a medication or have it as an inclusion criterion for the study population. In studies where it was included, the most frequent therapeutic group (23%) was antineoplastic and immunomodulatory agents. Pharmacological interventions were the most commonly evaluated (41%) followed by surgical (21%). Three out of four controls used were usual care or pharmacological treatment.

Table 2 shows methodological information on RWD. The main source of RWD costs and outcomes was information systems, such as the use of administrative databases. Most frequent sample size was between 100 and 1000. Some 83% of studies were not randomized. The most frequently mentioned potential bias was confounding selection and the most common forms of control described were sensitivity analysis and propensity score.

Table 3 shows methodological aspects related to the economic evaluation. All studies employed direct costs while 15% combined direct and indirect. Mean survival measured as Life-years, life-years gained, life-years or mortality was the most frequently used clinical outcome in 46% of studies, while only 17% can be considered cost-utility evaluations. Around 50% of studies used a time horizon between 1 and 5 years. A total of 41% of studies carried out the evaluation from the payer’s perspective.

Supplementary Material 2 and 3 show general characteristics, aspects of RWD and information on the economic evaluation in each study.

**Figure 2:**
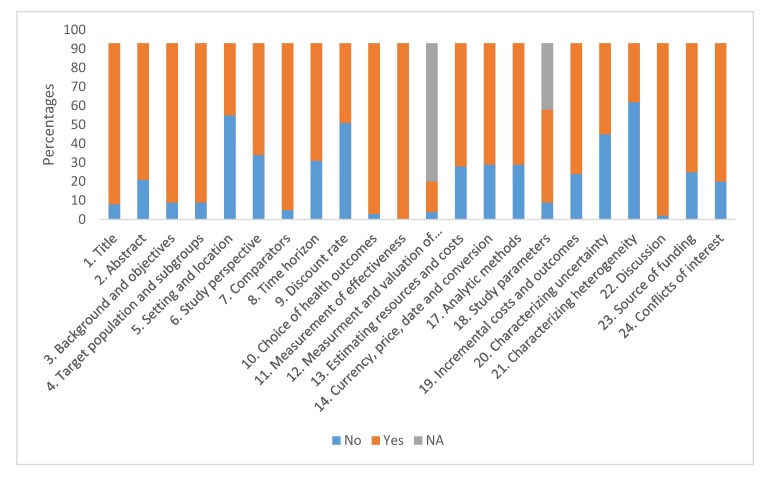
Quality assessment according CHEERS check list. Items 15 (Choice of model) and 16 (assumptions) only apply to mathematical models and were not scored. NA: Not apply

The methodological quality of the economic evaluation for each included study is shown in Figure 2. It can be seen that the study items most frequently fulfilled with respect to the CHEERS checklist were the title, study population/subgroup, comparators, selection of clinical results and discussion. The least frequently fulfilled were setting and location, discount rate and characterizing heterogeneity. Of the items evaluated, two were not applicable in 100% of the reviewed studies as they are items on decision models (items 15 and 16) and item 12 did not apply to economic evaluation studies that did not use QALY as a measure of effectiveness. Forty-four studies (47.31%) met at least 80% of the CHEERS criteria (17 or more items, not including items 12, 15 and 16) and 5 (5%) met at least 50% of checklist items.

The CHEERS checklist focuses on the methodological aspects of EE rather than RWD. However, we examined whether there was any relationship between studies that met CHEERS criteria and RWD aspects, such as sample size, study randomization or whether the authors applied bias control methodology or not. This information is shown in Table 4, where it can be seen that most studies with a sample size lower than 1000 met at least 80% of CHEERS criteria, while 60% of those with 1000 or more reached the same threshold. Randomized studies and those that reported bias control met the highest percentage of CHEERS criteria (80% or more).

Please see Supplementary Material 4 for details on study quality assessments.

## 4. Discussion

An assessment was conducted of the methodological quality of the economic evaluations in the 93 studies using RWD. Pharmacological interventions were the most frequently evaluated. The most commonly used clinical outcome was survival. Approximately half of the studies used a time horizon of 1–5 years and carried out the evaluation from the funding entity’s perspective. Roughly half of the studies met 80% of CHEERS criteria. The most frequently fulfilled items were discussion and measurement of clinical effectiveness and the least were setting and location and characterizing heterogeneity.

Some studies did not specify in which country or setting the EE was performed, which is an important aspect for decision-makers or researchers who would like to use the study as a basis. As the decision to apply the results of an EE study is used for inclusion and or reimbursement, it will depend on other aspects of the context in which it is carried out.

A systematic review of the use of RWD for EE in Germany was identified [111] and some comparisons with this are presented below. However, some inclusion criteria differ from those of the present study, such as studies that use routine data as a source of information for costs or effects or both. Our review includes studies that use RWD in costs and effects. Moreover, Gansen [111] only included studies based on data from Germany and the authors put no restrictions on the publication language while, in contrast, we placed no restrictions on specific country databases but did restrict the publication languages.

Gansen [111] found that the principal illnesses assessed were cardiovascular diseases followed by type II diabetes mellitus whereas neoplasms were the main focus of evaluation in our review. This may be due to the fact that Gansen [111] only reviewed studies performed in Germany. Regarding the higher frequency of evaluations related to neoplasm interventions, it may be because some, such as lung cancer, are among the most frequently diagnosed and cause the most mortality [112,113]. Approximately 50% of the EE studies on neoplasm interventions were conducted in Canada (89% of studies in this country were on this illness group) and the United States (40% of total illnesses).

QALYs were used as a measure of effectiveness in only 17% of studies, despite being the health outcome recommended by EE methodological guidelines [114,115], especially in illnesses that compromise quality of life, such as cancer, musculoskeletal diseases or mental disorders. Furthermore, QALYS are a useful measure of effectiveness as they allow comparison between interventions in distinct illnesses. Nevertheless, use of QALYs in the EE reviewed was higher than in the Gansen [111] studies, which identified only two studies using this outcome (5.7%). It is possible that one of the reasons why QALYs were not included in EE using RWD is because quality of life is not systematically recorded in health systems or that the utilities cannot be estimated with registered clinical outcomes.

To ensure quality in an EE study based on RWD in analysis of cost and outcomes variables, it is important to have a good source of information for use of RWD. Quality will depend on the degree of data reliability and how to use it to respond to specific questions [5]. A crucial aspect is sample size, which in the reviewed studies varied between less than 100 and more than 5000. Sample size affects results, so it is vital to determine what range of sample size ensures that the economic evaluation has sufficient validity.

Study randomization is another key issue in use of RWD where the potential risk of bias is the most significant limitation that can arise in this type of data [5]. In this review, there was a predominance of non-randomized studies as sources of RWD were mainly information systems, where randomization is not possible in retrospective studies. However, some studies reported using methods to control bias or the risk of bias. Nevertheless, despite the application of statistical approaches to adjust for selection bias in observational studies, they do not have the methodological rigor of RCTs [5] that favor internal validity but are easily generalized to a more heterogeneous population [4,116]. More critical are those studies that did not report biases or methods to control them which, in this review, totaled approximately 20 studies.

The relationship between quality of RWD and EE methodological quality is reflected in the results of this review where studies with sample sizes of 1000 or more, randomized and with bias control, met more CHEERS criteria.

Only one study was performed in two countries [75] which evaluated the effectiveness and costs of an intervention. This may be due to the complexity of harmonizing data from differing health and registration systems.

Temporal horizon is a fundamental aspect in EE. However, we observed that some studies did not report it, or if they did, its use was not justified. Very few studies employed the society perspective, which is the most extensive [1] and, depending on the EE, the most appropriate, as there are costs borne by families that could represent 30% of total costs [115]. To approach the study from the society perspective, information on costs is required which is not normally recorded as, in most cases, the sources of information were administrative databases and/or medical records. In common with the Gansen review, most studies employed the payer’s perspective. Furthermore, some studies did not specify the study’s perspective.

Regarding the results of the methodological quality assessment, study strengths (items marked “yes” for 80% or more of the studies reviewed) were: proper placement of the title; defined aims, study population and comparators; description of health-outcome selection; measurement of effectiveness; and an adequate discussion section. Areas for improvement were also identified (items marked “no” for 50% or more of studies reviewed). For instance, half of the studies did not specify relevant aspects of decision-making procedures. EE setting and location can greatly affect results. Approximately half of the studies reported a discount rate and very few characterized heterogeneity, that is, they did not explain whether differences in costs, effects or cost-effectiveness were due to variations between patient subgroups or other observed variability. Heterogeneity is of great relevance in studies that use RWD as the patients included do not usually meet strict inclusion criteria or, at least, these are not defined prior to their participation. Consequently, discussion of possible differences due to distinct subject characteristics is crucial.

Approximately half of the studies fulfilled 80% of checklist items. This is in line with results reported by Gansen [111], who also identified characterization of heterogeneity as a weakness; although, in contrast with this review, the German review identified other weaknesses, such as not reporting the discount rate (item no. 9), currency, price date and conversion, and characterization of uncertainty.

As reflected in this review and the one conducted in Germany, use of RWD in EE is increasing [111]. RWD are an essential source for coverage, funding and health-technology reimbursement decisions [5]. However, as mentioned previously, these studies have some methodological limitations, such as confounding selection, identified by the authors of the studies in this review as the main potential risk. In another review [2], the main biases were confounding and missing data. A further risk in this type of information, frequent but not detailed, is registration quality. Registration accuracy and the heterogeneity of each variable considered as health service use should be reflected.

These RWD limitations represent an important methodological challenge as the benefits of RWD with respect to RCTs. These designs should be complementary given that RCTs continue to be the standard for demonstrating the clinical efficacy of interventions. However, it is necessary to determine their effectiveness through long-term observational studies, pragmatic studies and use of administrative databases [5].

Assessment by independent pairs of researchers and methodological rigor were applied throughout the review. In cases of discrepancies, consensus was sought and if agreement was not reached, a third researcher made the decision. We hope that the results obtained contribute information on the methodological quality of the evaluated studies and are useful to decision-makers in terms of recognizing good quality studies on health technology. Nevertheless, this review has some limitations.

One limitation identified was ambiguity in interpreting the checklist used to evaluate EE methodological quality, especially on some items such as CHEERS item 18 which refers to study parameters. Conducting the review using independent pairs facilitated identification of the ambiguities and increased the likelihood of the results being transparent and comparable over time [117]. In addition, the researchers came from different disciplines (pharmacy, medicine and economics) which allowed a comprehensive view and was a strength of the study.

Another limitation was that only studies in English or Spanish were included, a restriction which led to the omission of published studies that met other inclusion criteria.

The methodological quality of the performance of the RWD studies was not assessed, although the one reported by the authors of the reviewed studies was considered valid. There are various checklists available to evaluate the quality of RWD studies. One is that proposed by the ISPOR Task Force [6], which consists of 7 items related to Hypothesis Evaluating Treatment Effectiveness (HETE). As noted in the systematic review protocol registered at Figshare, the intention was to use this checklist with the studies included. However, although we largely adhered to the protocol, RWD quality was not assessed with this checklist as the items seemed ambiguous and, as mentioned above, some items appeared on the CHEERS list, so it was decided to use the CHEERS one only. The possible reason for the non-tracking of the ISPOR Task Force checklist is its publication date, as most articles were published prior to this date.

Another checklist is that proposed by Kreif, which consists of 5 questions dealing with some statistical aspects [118]. Nevertheless, we did not use it as, although it was complementary, it did not substitute the EE checklist and had some limitations such as not covering some aspects of statistical analysis. Moreover, this checklist did not allow a description of which specific statistical method should be prescribed to approach selection bias in analysis of cost-effectiveness [2].

For future studies, it would be beneficial to have a validated, adapted tool available to assess the methodological quality of EE studies using RWD. Health Technology Assessment agencies in Europe could collaborate with RWD use policies and recommendations on practical aspects of RWD collection and analysis [7]. These policies could act as a basis for the use of RWD in EE. This standardization is essential because, as the results of this review showed, the use of RWD to create Real World Evidence is increasing. Despite its limitations, the use of this evidence is becoming more and more necessary. In the future, its limitations should be considered and compensated. This evidence will be crucial in enabling improvements in the quality, safety and value of health care.

## 5. Conclusions

This review demonstrates that the use of RWD in carrying out EE with individual patient data is an increasingly common practice. A total of 93 studies were identified that met these conditions and their methodological quality was assessed using the CHEERS checklist. It was observed that fewer than half of the studies fulfilled 80% of checklist criteria. It shows the low quality of the EE included in this review. More attention should be paid to the reporting of methodologies and results in EE.

Meeting CHEERS checklist criteria is associated with RWD methodological aspects; studies with samples sizes of 1000 or more, randomized studies and those that reported some method of controlling bias were those that had the greatest likelihood of fulfilling 80% of CHEERS criteria.

Some important methodological differences were noted in RWD use and in the presentation of results, which represents a considerable challenge regarding standardization of methodology in EE using RWD. Meeting this challenge would facilitate decision-making among policy makers.

Use of the CHEERS checklist showed that there are important aspects of RWD that are not considered and that the use of more than one checklist is neither practical nor efficient and, as such, it would be valuable to have an EE checklist that includes RWD available.

## Figures and Tables

**Table 1:** Characteristics of studies included.

Characteristics	n (%)
Year publication	
1995–2010	6 (6.45)
2011–2015	59 (63.44)
2016–2018	28 (30.11)
Region/Country	
Europe	22 (23.66)
United States	20 (21.51)
China	11 (11.83)
Canada	9 (9.68)
Australia	4 (4.3)
Others	6 (6.45)
Not reported	16 (17.20)
Setting	
Hospital	45 (48.39)
Primary care	15 (16.13)
Hospital and primary care	3 (3.23)
Specialized care	3 (3.23)
Not reported	27 (29.03)
Source of funding	
Public	42 (45.16)
Private	15 (16.13)
Public and private	3 (3.23)
No funding	4 (4.3)
Not reported	29 (31.18)
Eligibility criteria based on disease (ICD10)	
Chapter II: Neoplasms	35 (37.63)
Chapter IV: Endocrine, nutritional and metabolic diseases	10 (10.75)
Chapter IX: Diseases of the circulatory system	14 (15.05)
Chapter XIII: Diseases of the musculoskeletal system and connective tissue	10 (10.75)
Others	24 (25.81)
No disease	1 (1.08)
Eligibility criteria based on treatment	
ATC L (antineoplastic and immunomodulating agents)	22 (23.66)
Other ATC codes (A, B, C, J, N and R)	14 (15.05)
Radiotherapy	2 (2.15)
Cellular therapy	1 (1.08)
Not reported/not applied	54 (58.06)
Intervention	
Pharmacological	38 (40.86)
Surgical	20 (21.51)
Screening program	9 (9.68)
Prevention program (education)	6 (6.45)
Management program	7 (7.53)
Rehabilitation	4 (4.3)
Radiotherapy	3 (3.23)
Cellular therapy	2 (2.15)
Others	4 (4.3)
Control	
Usual care	38 (40.86)
Pharmacological	29 (31.18)
Surgical	17 (18.28)
Screening program	3 (3.23)
Others	6 (6.45)

ICD: International Statistical Classification of Diseases; ATC: Anatomical therapeutic clinical.

**Table 2:** Methodological information about Real World Data (RWD).

Characteristics	n (%)
RWD source	
Information systems	63 (67.74)
Clinical records	12 (12.9)
Clinical records and information systems	11 (11.83)
Not reported	7 (7.53)
Sample size	
<100	9 (9.68)
Between 100 and 1000	38 (40.86)
Between 1000 and 5000	24 (25.81)
>5000	18 (19.35)
Not reported	4 (4.3)
Study methodology	
Not randomized	77 (82.8)
Randomized	16 (17.2)
Reported potential risk of bias *	
Confounding selection	39 (41.94)
Confounding factors	14 (15.05)
Adjustment variables not specified	14 (15.05)
Missing data	10 (10.75)
Bias in measurement or registry	10 (10.75)
Censoring data	3 (3.23)
Time-related bias	2 (2.15)
Not reported	19 (20.43)
Reported methods to control *	
Sensitivity analysis	27 (29.03)
Propensity score	25 (26.88)
Bootstrap	20 (21.51)
Adjusting by confounding	6 (6.45)
Imputation missing data	5 (5.38)
Not reported	22 (23.66)

* Some studies reported different potential risks of bias or methods to control.

**Table 3:** Methodologic information about economic evaluation.

Characteristics	n (%)
Costs/inputs	
Direct costs	79 (84.95)
Direct and indirect costs	14 (15.05)
Clinical outcomes/outputs *	
Life years (LYS, LYG, mortality)	43 (46.24)
Quality-adjusted life years (QALY)	16 (17.2)
Achievement of therapeutic objectives (LDL, Ha1C, viral response, etc.)	9 (9.68)
Events avoided	8 (8.6)
Cases diagnosed	6 (6.45)
Others **	21 (22.58)
Time horizon ***	
<1 year	18 (19.35)
Between 1 and 5 years	42 (45.16)
>5 years	14 (15.05)
Lifetime	3 (3.23)
Not reported	18 (19.35)
Perspective ****	
Payer	38 (40.86)
Health system	20 (21.51)
Hospital	8 (8.6)
Society	4 (4.3)
Other	2 (2.15)
Not reported	23 (24.73)

* Some studies reported different clinical outcomes, ** Successful therapeutic or surgical programs, health questionnaires or quality of life, *** Two studies used 2 different time horizons, **** Two studies used different perspectives.

**Table 4:** Relation between CHEERS and RWD.

Characteristics	Meet Less Than 80% of Criteria * n (%)	Meet 80% or More Criteria * n (%)
CHEERS	51 (54.84)	42 (45.16)
Sample size		
<100	6 (11.76)	3 (7.14)
Between 100 and 1000	27 (52.94)	11 (26.19)
Between 1000 and 5000	9 (17.65)	15 (35.71)
>5000	8 (15.69)	10 (23.81)
Not reported	1 (1.96)	3 (7.14)
Randomized		
Yes	7 (13.73)	9 (21.43)
No	44 (86.27)	33 (78.57)
Reported control bias		
Yes	31 (60.78)	41 (97.62)
No	20 (39.22)	1 (2.38)

* 17 or more criteria (of 21, excluding those that did not apply 15, 16) or that in the great majority did not apply (12).

## Supplementary Materials

The following are available online at https://www.mdpi.com/1660-4601/17/4/1171/s1, Supp-Material-1-Search- strategy; Suppl-Material-2-Charact; Suppl-Material-3-RWD-EE; Suppl-Material-4-CHEERS.
