# Supplementary material for: Economic Evaluations Informed Exclusively by Real World Data: A Systematic Review
Source: Int J Environ Res Public Health. 2020 Feb 12;17(4):1171. doi: 10.3390/ijerph17041171 (PMC7068655; doi:10.3390/ijerph17041171)
Supplement: Supplementary file 1 [file ijerph-17-01171-s001.zip › Suppl-Material-3-RWD-EE.docx]

**Supplementary material 3. RWD and EE characteristics of studies included**
