# Supplementary material for: Economic Evaluations Informed Exclusively by Real World Data: A Systematic Review
Source: Int J Environ Res Public Health. 2020 Feb 12;17(4):1171. doi: 10.3390/ijerph17041171 (PMC7068655; doi:10.3390/ijerph17041171)
Supplement: Supplementary file 1 [file ijerph-17-01171-s001.zip › Suppl-Material-4-CHEERS.docx]

**Supplementary material 4. Methodological quality of studies. CHEERS**
