# Supplementary material for: Economic Evaluations Informed Exclusively by Real World Data: A Systematic Review
Source: Int J Environ Res Public Health. 2020 Feb 12;17(4):1171. doi: 10.3390/ijerph17041171 (PMC7068655; doi:10.3390/ijerph17041171)
Supplement: Supplementary file 1 [file ijerph-17-01171-s001.zip › Supp-Material-1-Search- strategy.docx]

**Complementary material 1**

**Search strategy**

**PUBMED SEARCH**

**#1:** "Cost-Benefit Analysis"[Mesh] OR “cost utility” OR “cost effectiveness” OR “cost benefit”

**#2:** "Electronic Health Records"[Mesh] OR "Registries"[Mesh] OR “real world” OR RW OR RWD OR “big data” OR “population based” OR “population study” OR “national database” OR “national databases” OR “administrative data” OR “administration data” OR “Electronic Medical Records” OR “Electronic Medical Record” OR “Medical Record, Electronic” OR “Medical Records, Electronic” OR “Record, Electronic Medical” OR “Records, Electronic Medical” OR “Electronic Health Record” OR “Health Record, Electronic” OR “Health Records, Electronic” OR “Record, Electronic Health” OR “Records, Electronic Health” OR “Medical Records, Computerized” OR “Medical Record, Computerized” OR “Computerized Medical Record” OR “Record, Computerized Medical” OR “Records, Computerized Medical” OR “Computerized Medical Records” OR “Registry” OR “Population Register” OR “Population Registers” OR “Register, Population” OR “Registers, Population” OR “Parish Registers” OR “Parish Register” OR “Register, Parish” OR “Registers, Parish”

**#3:** #1 AND #2

**EMBASE SEARCH**

**#1**,"'cost benefit analysis' OR 'cost effectiveness analysis' OR 'cost utility analysis'"

**#2**,"'clinical data repository' OR 'electronic medical record' OR 'electronic health record' OR 'electronic patient record' OR 'electronic medical record system' OR 'medical record review'" OR 'data warehouse' OR 'patient data'

**#3**,"'medical record review'"

**#4**,"'electronic health record certification'" OR 'meaningful use criteria'"

**#5**,"'disease registry'"

**#6**,"#2 OR #3 OR #4 OR #5"

**#7**,"#1 AND #6",

**WEB OF SCIENCE (WOS) SEARCH**

**#1**: “Analyses, Cost-Benefit” OR “Analysis, Cost-Benefit” OR “Cost-Benefit Analyses” OR “Cost Benefit Analysis” OR “Analyses, Cost Benefit” OR “Analysis, Cost Benefit” OR “Cost Benefit Analyses” OR “Cost Effectiveness” OR “Effectiveness, Cost” OR “Cost-Benefit Data” OR “Cost Benefit Data” OR “Data, Cost-Benefit” OR “Cost-Utility Analysis” OR “Analyses, Cost-Utility” OR “Analysis, Cost-Utility” OR “Cost Utility Analysis” OR “Cost-Utility Analyses” OR “Economic Evaluation” OR “Economic Evaluations” OR “Evaluation, Economic” OR “Evaluations, Economic” OR “Marginal Analysis” OR “Analyses, Marginal” OR “Analysis, Marginal” OR “Marginal Analyses” OR “Cost Benefit” OR “Costs and Benefits” OR “Benefits and Costs” OR “Cost-Effectiveness Analysis” OR “Analysis, Cost-Effectiveness” OR “Cost Effectiveness Analysis**”**

**#2: "**Electronic Health Records"[Mesh] OR "Registries"[Mesh] OR “real world” OR RW OR RWD OR “big data” OR “population based” OR “population study” OR “national database” OR “national databases” OR “administrative data” OR “administration data” OR (“Electronic Medical Records” OR “Electronic Medical Record” OR “Medical Record, Electronic” OR “Medical Records, Electronic” OR “Record, Electronic Medical” OR “Records, Electronic Medical” OR “Electronic Health Record” OR “Health Record, Electronic” OR “Health Records, Electronic” OR “Record, Electronic Health” OR “Records, Electronic Health” OR “Medical Records, Computerized” OR “Medical Record, Computerized” OR “Computerized Medical Record” OR “Record, Computerized Medical” OR “Records, Computerized Medical” OR “Computerized Medical Records”) OR (“Registry” OR “Population Register” OR “Population Registers” OR “Register, Population” OR “Registers, Population” OR “Parish Registers” OR “Parish Register” OR “Register, Parish” OR “Registers, Parish”)

**#3:** #1 AND #2

**Centre for Review and Dissemination (CRD) SEARCH**

**1**: ((Analyses, Cost-Benefit) OR (Analysis, Cost-Benefit) OR (Cost-Benefit Analyses)) and ((Economic evaluation: ZTD and Abstract: ZPS) OR Full publication record: ZDT) IN NHSEED, HTA

**2**: ((Cost Benefit Analysis) OR (Analyses, Cost Benefit) OR (Analysis, Cost Benefit)) and ((Economic evaluation: ZTD and Abstract: ZPS) OR Full publication record: ZDT) IN NHSEED, HTA

**3:** ((Cost Benefit Analyses) OR (Cost Effectiveness) OR (Effectiveness, Cost)) and ((Economic evaluation: ZTD and Abstract: ZPS) OR Full publication record: ZDT) IN NHSEED, HTA

**4**: ((Cost-Benefit Data) OR (Cost Benefit Data) OR (Data, Cost-Benefit) and ((Economic evaluation: ZTD and Abstract: ZPS) OR Full publication record: ZDT) IN NHSEED, HTA

**5**: ((Cost-Utility Analysis) OR (Analyses, Cost-Utility) OR (Analysis, Cost-Utility)) and ((Economic evaluation: ZTD and Abstract: ZPS) OR Full publication record: ZDT) IN NHSEED, HTA

**6**: ((Cost Utility Analysis) OR (Cost-Utility Analyses) OR (Economic Evaluation)) and ((Economic evaluation: ZTD and Abstract: ZPS) OR Full publication record: ZDT) IN NHSEED, HTA

**7**: ((Economic Evaluations) OR (Evaluation, Economic) OR (Evaluations, Economic)) and ((Economic evaluation: ZTD and Abstract: ZPS) OR Full publication record: ZDT) IN NHSEED, HTA

**8**: ((Marginal Analysis) OR (Analyses, Marginal) OR (Analysis, Marginal)) and ((Economic evaluation: ZTD and Abstract: ZPS) OR Full publication record: ZDT) IN NHSEED, HTA

**9**: ((Marginal Analyses) OR (Cost Benefit) OR (Costs and Benefits) and ((Economic evaluation: ZTD and Abstract: ZPS) OR Full publication record: ZDT) IN NHSEED, HTA

**10**: ((Benefits and Costs) OR (Cost-Effectiveness Analysis) OR (Analysis, Cost-Effectiveness)) and ((Economic evaluation: ZTD and Abstract: ZPS) OR Full publication record: ZDT) IN NHSEED, HTA

**11**: ((Cost Effectiveness Analysis**))** and ((Economic evaluation: ZTD and Abstract: ZPS) OR Full publication record: ZDT) IN NHSEED, HTA

**12**: #1 OR #2 OR #3 OR #4 OR #5 OR #6 OR #7 OR #8 OR #9 R #10 OR #11

**13:** ((Electronic Health Records) OR (Registries) OR (real world)) and ((Economic evaluation: ZTD and Abstract: ZPS) OR Full publication record: ZDT) IN NHSEED, HTA

**14:** ((RW) OR (RWD) OR (big data)) and ((Economic evaluation: ZTD and Abstract: ZPS) OR Full publication record: ZDT) IN NHSEED, HTA

**15**: ((population based) OR (population study) OR (national database)) and ((Economic evaluation: ZTD and Abstract: ZPS) OR Full publication record: ZDT) IN NHSEED, HTA

**16**: ((national databases) OR (administrative data) OR (administration data)) and ((Economic evaluation: ZTD and Abstract: ZPS) OR Full publication record: ZDT) IN NHSEED, HTA

**17**: ((Electronic Medical Records) OR (Electronic Medical Record) OR (Medical Record, Electronic)) and ((Economic evaluation: ZTD and Abstract: ZPS) OR Full publication record: ZDT) IN NHSEED, HTA

**18**: ((Medical Records, Electronic) OR (Record, Electronic Medical) OR (Records, Electronic Medical) and ((Economic evaluation: ZTD and Abstract: ZPS) OR Full publication record: ZDT) IN NHSEED, HTA

**19**: ((Electronic Health Record) OR (Health Record, Electronic) OR (Health Records, Electronic)) and ((Economic evaluation: ZTD and Abstract: ZPS) OR Full publication record: ZDT) IN NHSEED, HTA

**20**: ((Record, Electronic Health) OR (Records, Electronic Health) OR (Medical Records, Computerized) and ((Economic evaluation: ZTD and Abstract: ZPS) OR Full publication record: ZDT) IN NHSEED, HTA

**21**: ((Medical Record, Computerized) OR (Computerized Medical Record) OR (Record, Computerized Medical) and ((Economic evaluation: ZTD and Abstract: ZPS) OR Full publication record: ZDT) IN NHSEED, HTA

**22**: ((Records, Computerized Medical) OR (Computerized Medical Records”) OR (Registry)) and ((Economic evaluation: ZTD and Abstract: ZPS) OR Full publication record: ZDT) IN NHSEED, HTA

**23**: ((Population Register) OR (Population Registers) OR (Register) and ((Economic evaluation: ZTD and Abstract: ZPS) OR Full publication record: ZDT) IN NHSEED, HTA

**24**: ((Population) OR (Registers, Population) OR (Parish Registers)) and ((Economic evaluation: ZTD and Abstract: ZPS) OR Full publication record: ZDT) IN NHSEED, HTA

**25**: ((Parish Register) OR (Register, Parish) OR (Registers, Parish)) and ((Economic evaluation: ZTD and Abstract: ZPS) OR Full publication record: ZDT) IN NHSEED, HTA

**26**: #13 OR #14 OR #15 OR #16 OR #17 OR #18 OR #19 OR #20 OR #21 OR #22 OR #23 OR #24 OR #25

**27**: #12 AND #26
